# Diagnostic Value and Safety of Emergency Single-Balloon Enteroscopy for Obscure Gastrointestinal Bleeding

**DOI:** 10.1155/2019/9026278

**Published:** 2019-08-26

**Authors:** Yipin Liu, Weiwei Jiang, Guoxun Chen, Yanqing Li

**Affiliations:** ^1^Department of Gastroenterology, Qilu Hospital, Shandong University, Jinan, Shandong Province 250012, China; ^2^Department of Gastroenterology, Yantai Affiliated Hospital of Binzhou Medical University, Yantai, Shandong Province 264000, China; ^3^Department of Nutrition, University of Tennessee, Knoxville, Tennessee 37909, USA

## Abstract

**Background:**

This study assesses the diagnostic performance of emergency single-balloon enteroscopy (SBE) for obscure gastrointestinal bleeding (OGIB) under general anesthesia versus conscious sedation.

**Study:**

The data of 102 OGIB in-patients from June 2015 to June 2018 were retrospectively analyzed. The diagnosis and detection rates and adverse events were calculated overall and in relation to age, gender, type of operation and anesthesia, bleeding type, different times of examination, and SBE route. All statistical analyses were performed using SPSS 24.0, and the diagnosis and detection rates were compared using the Chi-square test.

**Results:**

Among the 102 patients, 66 patients had positive findings, while 11 patients had suspected positive findings, and the diagnosis and detection rates were 64.7% and 75.5%, respectively. Ulcers (19.6%) and tumors (16.7%) were the most common causes of OGIB. There were no statistical differences in diagnosis and detection rates between the ages of ≥60 and <60 and between different genders. Patients with emergency SBE had higher diagnosis and detection rates (68.6% *vs.* 35.3%, *P* = 0.023; 80.0% *vs.* 47.1%, *P* = 0.016, respectively), when compared with nonemergency SBE patients. The diagnosis rate at 24 hours was higher than that at 2-7 days and one week (88.0% *vs.* 61.5%, *P* = 0.030; 88.0% *vs.* 53.8%, *P* = 0.007). For overt bleeding, the difference in diagnosis rates at 24 hours, 2-7 days, and one week was statistically significant (100.0% *vs.* 57.1%, *P* = 0.006; 100.0% *vs.* 57.1%, *P* = 0.006). For occult bleeding, the pairwise comparison revealed no statistical difference. Patients with general anesthesia had a higher detection rate, when compared to patients with conscious sedation (87.9% *vs.* 63.9%, *P* = 0.004). In addition, adverse events under general anesthesia were lower, when compared to adverse events under conscious sedation (28.8% *vs.* 69.4%, *P* = 0.020). There was no significant difference in adverse events at the different time points (*P* > 0.05).

**Conclusion:**

Emergency SBE under general anesthesia achieves higher diagnosis and detection rates, and fewer adverse events under conscious sedation, when compared to nonemergency SBE, regardless of the route. For patients with overt bleeding, it is easier to find lesions by emergency SBE within 24 hours.

## 1. Introduction

Obscure gastrointestinal bleeding (OGIB) is defined as recurrent or persistent gastrointestinal (GI) bleeding, the cause of which cannot be explained by investigations, such as esophagogastroduodenoscopy (EGD), colonoscopy, or radiographic imaging of the small intestine [1–3]. This is one of the most common critical diseases that accounts for 5-10% of gastrointestinal bleeding cases [4, 5]. Bleeding from the small intestine, called small-bowel bleeding, is the most common cause of OGIB.

The diagnosis of small-bowel bleeding has always remained challenging. Video capsule endoscopy (VCE) and device-assisted enteroscopy (DAE) are the first-line procedures for diagnosing small-bowel bleeding. VCE is safe, efficient, and noninvasive but presents with difficulties in localizing the lesion site and in the inability to perform biopsy and treat the lesion on site. DAE has the advantages of performing biopsies, repeated observation, polypectomy, drug injection, and other surgical operations [6–9]. DAE encompasses double-balloon enteroscopy (DBE) and single-balloon enteroscopy (SBE), which was developed based on DBE. SBE retains the advantages of DBE but has a more flexible mirror body, thereby providing greater vision, when compared to DBE [10, 11]. With a caliber similar to that of a standard upper endoscope, SBE has more than twice its length (200 cm), making it possible to perform most endoscopic diagnostic and therapeutic procedures [5]. In addition, the average preparation time for SBE instruments is significantly shorter than that of DBE [12]. Indeed, SBE has increasingly been used over the decade since Tsujikawa et al. first reported this in 2008 [13].

Recently, SBE has been used as an emergency tool for diagnosing OGIB under general anesthesia or conscious sedation [1, 11], with diagnosis rates ranging from 60% to 80% [10, 11, 14]. The European Society for Gastrointestinal Endoscopy recommended capsule endoscopy and balloon-assisted enteroscopy as first-line options for patients with persistent OGIB as the dominant symptom [15, 16]. Obviously, OGIB lesions are more likely to be identified with emergency SBE in patients during acute onset with blood loss. To date, there is no recommendation on the time at which SBE examination should be performed for dominant OGIB, but it has been generally considered that early endoscopic examination should be performed to treat persistent bleeding and improve the diagnosis and intervention rates [17–20]. This emphasizes the necessity and importance of emergency SBE.

Adverse events and complications have been reported in patients with general anesthesia or conscious sedation [5]. Movements due to discomfort/pain under conscious sedation might terminate the procedure. The onset of hypotension, hypoxia saturation, and apnea under general anesthesia can be horrible. Although most of the incidences can be rapidly and perfectly managed with the advances in medical care, the guarantee of sedation safety is imperative. The choice of sedation might play a vital role in the diagnostic value and safety of emergency SBE for OGIB. Therefore, the aim of the present study was to assess the diagnostic value and safety of emergency SBE for OGIB under general anesthesia *vs*. conscious sedation. In addition, the timing selection of emergency SBE was also assessed, since there are no clear guidelines on the timing for emergency SBE for patients with OGIB, at present. Furthermore, the diagnosis and detection rates and adverse events were assessed in patients who underwent colonoscopy at different time points.

## 2. Materials and Methods

### 2.1. Patients

The present study conducted a retrospective analysis of the data obtained from patients diagnosed with OGIB, who were admitted in the Department of Gastroenterology of Yantai Affiliated Hospital of Binzhou Medical University from June 2015 to June 2018. The study protocol was approved by the Medical Ethics Committee of Yantai Affiliated Hospital of Binzhou Medical University.

The time to endoscopy (*i.e.*, the interval from hospital admission to endoscopy) following acute upper gastrointestinal bleeding (AUGIB) has been adopted by the National Institute for Health and Clinical Excellence (NICE) [21], the Joint Advisory Group on Gastrointestinal Endoscopy (JAG) [22], and the European Society of Gastrointestinal Endoscopy (ESGE) [23] as a quality standard for both patients and endoscopy units. The NICE and ESGE recommend early endoscopy (<24 hours within admission) for all patients admitted with suspected AUGIB [21, 23]. Accordingly, the patients were divided into two major groups: the emergency SBE group (50 cases within 24 hours) and the nonemergency SBE group (52 cases over 24 hours). In the emergency SBE group, there were 25 cases within 12 hours and 25 cases between 12 and 24 hours. In the nonemergency SBE group, there were 26 cases within 2-7 days and 26 cases with more than one week.

Patients who met the following criteria were included in the present study: (1) patients with recurrent or persistent gastrointestinal bleeding and (2) patients with an unclear cause of bleeding after the early investigation. The following patients were excluded from the present study [14]: (1) patients with gastrointestinal motility disorders; (2) patients with suspected small-bowel obstruction or intestinal fistula; (3) patients who were unable to finish the bowel preparation due to complete intestinal obstruction; (4) patients implanted with electromedical equipment or patients with a history of cardiopulmonary function; (5) patients with psychosis or dementia; (6) patients with high risk of anesthesia or abnormal coagulation function; (7) patients with a history of multiple abdominal surgeries; (8) patients with severe hepatic cirrhotic conditions; and (9) pregnant women.

These patients were subclassified into overt bleeding and occult bleeding. Overt bleeding was defined as recurrent or persistent hematochezia or melena, which was visible to the patient and physician. Occult bleeding was defined as recurrent or persistent anemia and having a positive fecal occult blood test. The hemoglobin values and clinical manifestations were both taken into consideration. Patients who had overt or occult bleeding within 24 hours after hospital admission underwent emergency SBE. Otherwise, nonemergency SBE was applied [24, 25]. All patients and/or their authorized family members provided a signed written informed consent for the SBE examination and treatment (if needed).

### 2.2. Bowel Preparation

Bowel preparation was required for patients undergoing nonemergency SBE *via* the anal route. Briefly, patients took a low residue diet one day before the examination, fasted for 8-10 hours, and took an intestinal cleanser compound, polyethylene glycol (Heshuang, Wanhe Pharmaceutical Co. Ltd., Shenzhen, China), at 6-10 hours before the examination. Bowel preparation was not performed for patients with acute massive GI bleeding, which was defined as a case that required the transfusion of at least four units of blood within 24 hours in the hospital or as hypotension with a systolic blood pressure of <90 mm Hg. Patients who received SBE *via* the oral route were required to undergo 12-hour fasting, but without bowel preparation.

### 2.3. SBE Procedures, Anesthesia, and Sedation

The SIF-Q260 SBE system of Olympus was used (Tokyo, Japan). The procedure was performed by an experienced endoscopic physician and an assistant. The SBE procedures were performed *via* either the oral or anal route. The choice of route was based on the clinical features and pre-DAE investigations, which provided a preliminary suggestion of the possible lesion site. Patients who did not have a positive result from the previous examinations were recommended to receive SBE *via* the oral route first, considering the intestinal anatomy. For patients who had a unilateral negative result (anal route or oral route), their small intestines were marked with methylene blue or Indian ink, and further examinations were performed from the other side.

Patients were given either conscious sedation or general anesthesia. Blood oxygen saturation and electrocardiography were dynamically observed during the procedure. For conscious sedation, patients were administered with fentanyl before the examination, with a loading dose of 50-100 *μ*g and continued with 0.833-2.083 *μ*g/h until the ideal level of mild to moderate sedation was achieved [26]. For general anesthesia, patients received an intravenous injection of propofol, with a loading dose of 1.5-2.5 mg/kg. The dose was adjusted, when needed, by adding 0.2-0.5 mg/kg according to the manifestations or movements of the patients, or maintained at 6-10 mg/(kg·h) to ensure that the patient was unconscious and immobile during the examination [27].

### 2.4. Assessment of Diagnostic Values and Safety

The diagnostic findings of the SBE were classified as positive, suspected positive, and negative. A positive finding was defined when the bleeding cause was clearly identified by the examination [27]. A suspected positive finding was defined when the bleeding cause was not fully explained by the examination and requires an endoscopic diagnosis [20]. A negative finding was defined when no abnormality was detected by the examination [28].

Vital signs and adverse events were closely monitored and recorded during the SBE procedure under general anesthesia or conscious sedation.

### 2.5. Statistical Analysis

Data were expressed as mean ± standard deviation. All statistical analyses were performed using SPSS 24.0, and comparisons of the diagnosis rate and detection rate were performed using the Chi-square test. A *P* value of <0.05 was considered statistically significant.

## 3. Results

### 3.1. Characteristics of Patients Who Underwent SBE Procedures

Among the 102 patients included in the present study, 46 patients were male and 56 patients were female, and the ages of these patients ranged within 14-83 years old (51.3 ± 10.2 years old). Emergency and nonemergency SBE procedures were performed on 50 and 52 patients, respectively, the procedures were performed in 37 and 65 patients through oral and anal routes, respectively, and 66 and 36 patients were under general anesthesia and conscious sedation, respectively (Table 1).

### 3.2. Diagnostic Findings of the SBE

Among the 102 patients, positive (*n* = 66, 64.7%), suspected positive (*n* = 11, 10.8%), and negative (*n* = 25, 24.5%) findings were obtained. Thus, the overall diagnosis rate and detection rate were 64.7% and 75.5%, respectively (Table 1).

### 3.3. Diagnostic Findings in Relation to Age and Gender

Patients who were ≥60 years old appeared to have a higher diagnosis rate (74.2% *vs.* 60.6%) and detection rate (83.9% *vs.* 71.8%), when compared to patients who were <60 years old, but the difference was not statistically significant. Furthermore, there was no significant change in diagnosis rate between male and female patients (67.4% *vs.* 62.5%, Table 2).

### 3.4. Diagnostic Findings Obtained by Emergency and Nonemergency SBE

Patients who underwent emergency SBE had a significantly higher diagnosis rate (80.0% *vs.* 57.7%, *P* = 0.015) and detection rate (90.0% *vs.* 69.2%, *P* = 0.010), when compared to the nonemergency group (Table 3).

The diagnosis rate was significantly higher when the procedure was performed within 12 hours after admission, when compared to procedures performed within 2-7 days or more than one week after admission (88.0% *vs.* 61.5%, *P* = 0.030 or 88.0% *vs.* 53.8%, *P* = 0.007). However, there was no significant difference in diagnosis rate among the other groups (Table 4).

### 3.5. Diagnostic Findings of Emergency and Nonemergency SBE in Relation to the Choice of Anesthesia and Sedation

The overall diagnosis rate and detection rate were significantly higher in the general anesthesia group than in the conscious sedation group (75.8% *vs.* 55.6%, *P* = 0.036; 87.9% *vs.* 63.9%, *P* = 0.004, respectively, Table 3).

### 3.6. Diagnostic Findings of Emergency and Nonemergency SBE in Relation to the Bleeding Pattern

Overall, 57 and 45 patients presented with overt and occult bleeding, respectively. Both the diagnosis rate (73.7% *vs.* 53.3%, *P* = 0.033) and detection rate (84.2% *vs.* 64.4%, *P* = 0.021) were significantly higher in patients with overt bleeding than in patients with occult bleeding (Table 5).

Among patients who underwent emergency SBE, a similar diagnosis rate and detection rate were obtained in the 29 patients with overt bleeding and 21 patients with occult bleeding. Among patients who underwent nonemergency SBE, 28 and 24 patients had overt and occult bleeding, respectively, with a diagnosis rate of 67.9% and 37.5%, respectively (*P* = 0.029), and a detection rate of 78.6% and 45.8%, respectively (*P* = 0.015) (Table 5).

For overt bleeding, the diagnosis rate was significantly higher when the procedure was performed within 12 hours or between 12-24 hours, when compared to the rate when the procedure was performed between 2 and 7 days or after more than one week (all, 100.0% *vs.* 57.1%, *P* < 0.01). However, for occult bleeding, there was no significant difference in diagnosis rate among groups with different time points (Table 6).

### 3.7. Diagnostic Findings of Emergency and Nonemergency SBE in Relation to the Insertion Route

Overall, 37 and 65 patients underwent the procedure *via* the oral route and anal route, respectively. For patients who underwent the procedure *via* the oral route, compared with nonemergency SBE, emergency SBE obtained a higher diagnosis rate (80.0% *vs.* 45.5%, *P* = 0.036) and detection rate (93.3% *vs.* 63.6%, *P* = 0.039). For patients who underwent the procedure *via* the anal route, emergency SBE had a higher diagnosis rate (77.1% *vs.* 50.0%, *P* = 0.023) and detection rate (88.6% *vs.* 66.7%%, *P* = 0.032), when compared to nonemergency SBE. Among patients who received emergency SBE and nonemergency SBE, there were no differences in diagnosis and detection rates between the two routes (Table 7).

### 3.8. Vital Signs and Adverse Events of Patients with General Anesthesia or Conscious Sedation

The vital signs, such as fluctuation range of respiration, heart rate, and systolic pressure, were similar between patients with general anesthesia and patients with conscious sedation. However, fewer adverse events were observed in the general anesthesia group than in the conscious sedation group (*P* = 0.020, Table 8).

There was no difference in adverse events among the SBE procedures performed at different time points after admission (Table 9).

## 4. Discussion

In the present study, SBE was performed on patients with OGIB, and it was found that for 102 patients, the diagnosis rate was 64.7% and the detection rate was 75.5%. This was in accordance with previous studies that reported diagnosis rates ranging within 41-73% [10, 11, 13, 29]. The most common causes of OGIB diagnosed by SBE were small intestine ulcers (19.6%), tumors (16.7%), vascular malformation (14.7%), and polyps (9.8%). In addition, 10.8% of OGIB cases were caused by nonspecific inflammation, intestinal focal erosion, diverticulum, and swelling.

A recent Chinese study conducted by Zhang et al. reported that inflammatory lesions/diseases accounted for most of the OGIB cases, followed by neoplasms [30].

However, the epidemiology of OGIB reported by Western countries was different from the present results, in which small intestine vascular disease accounted for most of the OGIB [31, 32], followed by ulcers and erosions [16]. In China, nonsteroidal anti-inflammatory drugs (NSAIDs) and oral anticoagulants (OACs) are more frequently used in adults and the elderly [33–35], which is one of the main causes of ulcers in the GI tract. Malignant tumors of the small intestine account for 2% of cancers of the digestive system, and the incidence of small intestine tumors has significantly increased in recent years [36]. In the present study, 16.7% of patients were diagnosed with small intestine tumors by SBE, which is 3- to 4-fold higher than that (2%-6%) reported in Western countries [37, 38].

In the present study, patients ≥60 years old had higher, albeit not significantly, diagnosis and detection rates, when compared to patients <60 years old, and the prevalence of ulcers was also higher in patients ≥60 years old, when compared to that in patients <60 years old. This observation can be explained by the fact that patients over 60 years old are more likely to receive NSAIDs or have other serious complications. In addition, in the present study, regardless of the type of sedation, the emergency SBE achieved significantly higher diagnosis and detection rates, when compared with nonemergency SBE, although there was no difference in the diagnosis rate between SBE procedures performed at 12 hours and at 12-24 hours. Furthermore, more lesions were detected at 12 hours and 12-24 hours, when compared to lesions detected at 2-7 days and after more than one week. This finding is conceivably due to the fact that patients undergoing emergency SBE have more emergent disease courses and more massive bleeding, when compared with those undergoing nonemergency SBE, thereby making it easier to be identified by the mirror of SBE.

Although SBE is relatively a noninvasive operation, the insertion of the mirror and tube may cause abdominal pain, distension, nausea, vomiting, and other discomfort while the patient is awake. In clinic, sedation is applied to provide the endoscopist a better operational environment [39–41]. Due to the advances in medical techniques, general anesthesia is safer than sedation and provides a more comfortable medical treatment. In the present study, although a similar diagnosis rate was achieved between the general anesthesia group and conscious sedation group, a higher detection rate was achieved in the general anesthesia group than in the conscious sedation group (*P* = 0.044). Patients who underwent emergency SBE had higher diagnosis and detection rates, when compared to patients who underwent nonemergency SBE, in both the general anesthesia and conscious sedation groups. In addition, all patients in the present study smoothly underwent the examination, without any severe complications. Therefore, SBE under general anesthesia is a safe and effective investigational method.

Massive bleeding in patients could cause hemorrhagic shock, circulatory dysfunction, and multiple organ failure. Performing early endoscopic examinations on patients with GI hemorrhage would increase the diagnosis rate and improve the intervention strategy [24]. The intestinal mucosal examination coverage ratio can reach more than 86% when DAE is performed *via* both the oral and anal route. The diagnosis rate for OGIB patients with overt bleeding has been reported to range within 41-73% [8, 9]. In the present study, it was found that the overt bleeding group had a diagnosis rate of 73.7%. Monkemuller et al. reported that through emergency DBE, it was easier to find bleeding lesions in patients with overt OGIB [24]. In the present study, through emergency SBE, patients with overt bleeding appeared to have higher diagnosis and detection rates, when compared to patients with occult bleeding, although the differences were not statistically significant. This was in agreement with the observation reported by Pinto-Pais et al., in which the positive results of the emergency SBE correlated with overt OGIB [42]. Furthermore, the study conducted by Rodrigues et al. revealed that the diagnostic and therapeutic impact of BAE was higher in an urgent setting [43]. Moreover, Chung et al. reported the highlights of the benefits of early deep enteroscopy for the treatment of small intestinal bleeding [44].

To date, there are no recommendations for SBE performing time in overt OGIB. It has been generally agreed that patients with persistent hemorrhage should be treated with early endoscopic examination, in order to improve the diagnosis and intervention rates [17, 44], although a study conducted by Nelson et al. reported that the diagnostic and therapeutic yields were not significantly different between the emergent (within 24 hours) and nonemergent procedures (greater than 24 hours) [45]. In the present retrospective study, it was found that there was no significant difference between overt bleeding at 12 hours and 12-24 hours, but there was a significant difference when compared with overt bleeding at 2-7 days and after more than one week. This suggested that it is easier to detect lesions by improving SBE within 24 hours in patients with dominant hemorrhage. In addition, there was no difference in adverse events between SBE tests at different time points (*P* > 0.05). This is consistent with a study [31] that suggested that emergency SBE examined within 24 hours after the onset of OGIB or within 24 hours after admission can lead to excellent diagnosis and safety.

The present study demonstrates that emergency SBE under general anesthesia has great diagnostic value for OGIB patients, because it provides clear images, operational accuracy, and a high detection rate. However, the disadvantage of emergency SBE under general anesthesia is that many contraindications exist. Furthermore, SBE cannot achieve the whole bowel examination in some patients. These disadvantages can be overcome by barium X-ray, angiography, nuclide scan, and VCE [46, 47].

In conclusion, intestinal ulcers account for the majority of OGIB cases, followed by tumors and vascular malformation. Emergency SBE under general anesthesia achieves higher diagnosis and detection rates, when compared to nonemergency SBE, under conscious sedation, regardless of the route. Furthermore, the diagnosis rates were higher in patients with overt bleeding than in patients with occult bleeding. Moreover, fewer adverse events were present in patients with general anesthesia, when compared to patients with conscious sedation.

## Figures and Tables

**Table 1:** Characteristics of patients who received single-balloon enteroscopy (SBE) procedures.

	Emergency (*n* = 50)	Nonemergency (*n* = 52)	Total (*n* = 102)
Gender			
Male	27 (54.0)	19 (36.5)	46 (45.1)
Female	23 (46.0)	33 (63.5)	56 (54.9)
Age			
<60 years old	18 (36.0)	13 (25.0)	31 (30.4)
≥60 years old	32 (64.0)	39 (75.0)	71 (69.6)
Anesthesia/sedation			
General anesthesia	31 (62.0)	35 (67.3)	66 (64.7)
Conscious sedation	19 (38.0)	17 (32.7)	36 (35.3)
Insertion route			
Oral	15 (30.0)	22 (42.3)	37 (36.3)
Anal	35 (70.0)	30 (57.7)	65 (63.7)
Endoscopic findings			
*Positive*	38 (76.0)^∗^	28 (53.8)	66 (64.7)
Ulcer	12 (24.0)	8 (15.4)	20 (19.6)
Jejunum	4	2	
Ileum	6	4	
Colon	2	2	
Tumor	9 (18.0)	8 (15.4)	17 (16.7)
Jejunal lymphoma	1	0	
Jejunal malignant stromal tumor (low degree)	3	1	
Jejunal malignant stromal tumor (high degree)	2	0	
End-colon cancer	1	0	
Colon adenocarcinoma	2	4	
Jejunal leiomyoma	0	1	
End ileal carcinoid	0	1	
Rectal carcinoid	0	1	
Vascular malformation	9 (18.0)	6 (11.5)	15 (14.7)
Polyp	5 (10.0)	5 (9.6)	10 (9.8)
Stale hemorrhage	2 (4.0)	0 (0.0)	2 (2.0)
Hemangioma	1 (2.0)	0 (0.0)	1 (1.0)
Parasite	0 (0.0)	1 (1.9)	1 (1.0)
*Suspected positive*	6 (12.0)	5 (9.6)	11 (10.8)
Nonspecific inflammation	3 (6.0)	2 (3.8)	5 (4.9)
Intestinal focal erosion	1 (2.0)	2 (3.8)	3 (3.0)
Diverticulum (no bleeding tendency)	1 (2.0)	1 (1.9)	2 (2.0)
Swelling (no bleeding tendency)	1 (2.0)	0 (0.0)	1 (1.0)
*Negative*	6 (12.0)	19 (36.5)	25 (24.5)

Data are expressed in number (%). ^∗^*P* < 0.05, compared with nonemergency single-balloon enteroscopy.

**Table 2:** Diagnostic findings in relation to age and gender.

Diagnostic findings	Age		Gender	
	≥60 (*n* = 31)	<60 (*n* = 71)	Male (*n* = 46)	Female (*n* = 56)
*Positive*	23 (74.2)	43 (60.6)	31 (67.4)	35 (62.5)
Ulcer	7 (22.6)	13 (18.3)	8 (17.4)	12 (21.42)
Tumor	6 (19.4)	11 (15.5)	9 (19.6)	8 (14.3)
Vascular malformation	5 (16.1)	10 (14.1)	7 (15.2)	8 (14.3)
Polyp	4 (12.9)	6 (8.5)	4 (8.7)	6 (10.7)
Stale hemorrhage	1 (3.2)	1 (1.4)	2 (4.3)	0 (0.0)
Hemangioma	0 (0.0)	1 (1.4)	1 (2.2)	0 (0.0)
Parasite	0 (0.0)	1 (1.4)	0 (0.0)	1 (1.8)
*Suspected positive*	3 (9.7)	8 (11.3)	5 (10.9)	6 (10.7)
Nonspecific inflammation	1 (3.2)	4 (5.6)	2 (4.3)	3 (5.3)
Intestinal focal erosion	1 (3.2)	2 (2.8)	1 (2.2)	2 (3.6)
Diverticulum (no bleeding tendency)	0 (0.0)	2 (2.8)	1 (2.2)	1 (1.8)
Swelling (no bleeding tendency)	1 (3.2)	0 (0.0)	1 (2.2)	0 (0.0)
*Negative*	5 (16.1)	20 (28.2)	10 (21.7)	15 (26.8)

Data are expressed in number (%).

**Table 3:** Diagnostic findings of emergency and nonemergency single-balloon enteroscopy in relation to the choice of anesthesia and sedation.

Diagnostic findings		Emergency		Nonemergency			Total		
	Total (*n* = 50)	GA (*n* = 31)	CS (*n* = 19)	Total (*n* = 52)	GA (*n* = 35)	CS (*n* = 17)	Total (*n* = 102)	GA (*n* = 66)	CS (*n* = 36)
Positive	40 (80.0)^∗^	26 (83.9)	14 (73.7)	30 (57.7)	24 (68.6)	6 (35.3)	70 (68.6)	50 (75.8)^#^	20 (55.6)
Suspected positive	5 (10.0)	4 (12.9)	1 (5.3)	6 (11.5)	4 (11.4)	2 (11.8)	11 (10.8)	8 (12.1)	3 (8.3)
Negative	5 (10.0)	1 (3.2)	4 (21.1)	16 (30.8)	7 (20.0)	9 (52.9)	21 (20.6)	8 (12.1)	13 (36.1)

GA, general anesthesia; CS, conscious sedation. Data are expressed in number (%). ^∗^*P* = 0.015, compared with nonemergency. ^#^*P* = 0.036, compared with conscious sedation.

**Table 4:** Diagnostic findings of emergency and nonemergency single-balloon enteroscopy at different time points after admission.

	Emergency			Nonemergency		
	12 hours (*n* = 25)	12-24 hours (*n* = 25)	Total (*n* = 50)	2-7 days (*n* = 26)	>1 week (*n* = 26)	Total (*n* = 52)
Positive	22 (88.0)^∗^^,#^	18 (72.0)	40 (80.0)	16 (61.5)	14 (53.8)	30 (57.7)
Suspected positive	2 (8.0)	3 (12.0)	5 (10.0)	5 (19.2)	1 (3.8)	6 (11.5)
Negative	1 (4.0)	4 (16.0)	5 (10.0)	5 (19.2)	11 (42.3)	16 (61.5)

Data are expressed in number (%). ^∗^*P* = 0.030, compared with 2-7 days. ^#^*P* = 0.007, compared with >1 week.

**Table 5:** Diagnostic findings of emergency and nonemergency single-balloon enteroscopy (SBE) in relation to the choice of anesthesia and sedation.

	Emergency SBE		Nonemergency SBE	
	Overt bleeding (*n* = 29)	Occult bleeding (*n* = 21)	Overt bleeding (*n* = 28)	Occult bleeding (*n* = 24)
*Positive*				
Overall	23 (79.3)	15 (71.4)^∗^	19 (67.9)^#^	9 (37.5)
GA	16 (55.2)	9 (42.9)	13 (46.4)	6 (25.0)
CS	7 (24.1)	6(28.6)	6 (21.4)	3 (12.5)
*Suspected positive*				
Overall	3 (10.3)	3 (14.3)	3 (10.7)	2 (8.3)
GA	2 (6.9)	1 (4.8)	2 (7.1)	1 (4.2)
CS	1 (3.4)	2 (9.5)	1 (3.6)	1 (4.2)
*Negative*				
Overall	3 (10.3)	3 (14.3)	6 (21.4)	13 (54.2)
GA	1 (3.4)	2 (9.5)	4 (14.3)	9 (37.5)
CS	2 (6.9)	1 (4.8)	2 (7.1)	4 (16.7)

GA, general anesthesia; CS, conscious sedation. Data are expressed in number (%). ^∗^*P* = 0.023, compared with nonemergency SBE for occult bleeding. ^#^*P* = 0.029, compared with occult bleeding among patients who received nonemergency single-balloon enteroscopy.

**Table 6:** Diagnostic findings of emergency and nonemergency single-balloon enteroscopy (SBE) at different time points after admission for overt and occult bleeding.

	Emergency SBE				Nonemergency SBE			
	12 hours (*n* = 25)		12-24 hours (*n* = 25)		2-7 days (*n* = 26)		>1 week (*n* = 26)	
	Overt bleeding (*n* = 14)	Occult bleeding (*n* = 11)	Overt bleeding (*n* = 15)	Occult bleeding (*n* = 10)	Overt bleeding (*n* = 14)	Occult bleeding (*n* = 12)	Overt bleeding (*n* = 14)	Occult bleeding (*n* = 12)
Positive	14 (100.0)^∗^	7 (63.6)	15 (100.0)^#^	6 (60.0)	8 (57.1)	6 (50.0)	8 (57.1)	6 (50.0)
Suspected positive	0 (0.0)	2 (18.2)	0 (0.0)	1 (10.0)	3 (21.4)	2 (16.7)	1 (7.1)	2 (16.7)
Negative	0 (0.0)	2 (18.2)	0 (0.0)	3 (30.0)	3 (21.4)	4 (33.3)	5 (35.7)	4 (33.3)

Data are expressed in number (%). ^∗^*P* = 0.006, compared with 2-7 days or >1 week. ^#^*P* = 0.004, compared with 2-7 days or >1 week.

**Table 7:** Diagnostic findings of emergency and nonemergency single-balloon enteroscopy (SBE) in relation to the insertion route.

	Emergency SBE		Nonemergency SBE	
	Oral (*n* = 15)	Anal (*n* = 35)	Oral (*n* = 22)	Anal (*n* = 30)
Positive	12 (80.0)^∗^	27 (77.1)^#^	10 (45.5)	15 (50.0)
Suspected positive	2 (13.3)	4 (11.4)	4 (18.2)	5 (16.7)
Negative	1 (6.7)	4 (11.4)	8 (36.4)	10 (33.3)

Data are expressed in number (%). ^∗^*P* = 0.036, compared with nonemergency single-balloon enteroscopy *via* the oral route. ^#^*P* = 0.023, compared with nonemergency single-balloon enteroscopy *via* the anal route.

**Table 8:** Adverse events in patients during emergency or nonemergency single-balloon enteroscopy under general anesthesia or conscious sedation.

Adverse event	Emergency SBE		Nonemergency SBE		Total	
	GA (*n* = 31)	CS (*n* = 19)	GA (*n* = 35)	CS (*n* = 17)	GA (*n* = 66)	CS (*n* = 36)
Overall	9 (29.0)	14 (73.7)	10 (28.6)	11 (64.7)	19 (28.8)	25 (69.4)^∗^
Agitation	2 (6.5)	2 (10.5)	2 (5.7)	2 (11.8)	4 (6.1)	4 (11.1)
Dizziness	2 (6.5)	2 (10.5)	3 (8.6)	2 (11.8)	5 (7.6)	4 (11.1)
Nausea/vomiting	1 (3.2)	3 (15.8)	2 (5.7)	2 (11.8)	3 (4.6)	5 (13.9)
Hypotension	3 (9.7)	1 (5.3)	2 (5.7)	1 (5.9)	5 (7.6)	2 (5.6)
Abdominal pain/bloating	1 (3.2)	6 (31.6)	1 (2.9)	4 (23.5)	2 (3.0)	10 (27.8)

GA, general anesthesia; CS, conscious sedation. ^∗^*P* = 0.020, compared between the two groups.

**Table 9:** Adverse events associated with the emergency or nonemergency single-balloon enteroscopy performed at different time points after admission.

Adverse event	Emergency SBE		Nonemergency SBE	
	12 hours (*n* = 25)	12-24 hours (*n* = 25)	2-7 days (*n* = 26)	>1 week (*n* = 26)
Overall	14 (56.0)	9 (36.0)	11 (30.6)	10 (38.5)
Agitation	3 (12.0)	1 (4.0)	2 (7.7)	2 (7.7)
Dizziness	3 (12.0)	2 (8.0)	2 (7.7)	2 (7.7)
Nausea/vomiting	1 (4.0)	2 (8.0)	3 (11.5)	2 (7.7)
Hypotension	3 (12.0)	2 (8.0)	1 (3.8)	1 (3.8)
Abdominal pain/bloating	4 (16.0)	2 (8.0)	3 (11.5)	3 (11.5)

Data are expressed in number (%).

## Data Availability

The datasets generated/analyzed during the present study are available from the corresponding authors on reasonable request.
